# Activation of MAPK and Cyclin D1/CDK4 in Malignant Transformation of Human Embryonic Lung Fibroblasts Induced by Silica and Benzopyrene

**DOI:** 10.31557/APJCP.2020.21.2.295

**Published:** 2020

**Authors:** Huan Wang, Shuyu Xiao, Yali Tang, Ke Han, Zheng Zhang, Yulan Jin, Fuhai Shen

**Affiliations:** 1 *Hebei Province Key Laboratory of Occupational Health and Safety for Coal Industry, School of Public Health, North China University of Science and Technology, No. 21 Bohai Road, Caofeidian District, *; 2 *Tangshan City Center for Disease Control and Prevention, 52 North Weiguo Road, Tangshan, Hebei Province, China. *

**Keywords:** Silica- B(a) P, HELF, MAPK, cyclin D1, CDK4

## Abstract

**Objective:**

Silica and Benzo(a)pyrene are listed as carcinogens. This study aims to explore Cyclin D1, CDK4 and difference of cell cycle adjusted by MAPK signal transduction pathway in silica and B(a)P-induced malignant transformation of human embryonic lung fibroblasts.

**Methods:**

Activity of the subfamily (ERK, p38 and JNK) of mitogen-activated protein kinase (MAPK), cyclin D1 and CDK4 (cyclin dependent kinase) were evaluated using Human embryonic lung fibroblast (HELF) purchased from the cell room, basic research institute, Chinese Academy of Medical Sciences. The expression of *cyclin D1* and *CDK4* (cyclin dependent kinase) were measured in silica and B(a)P induced malignant using Western blot (WB) assay.

**Results:**

*P-ERK* and *P-JNK* expression increased significantly (P<0.01), while *CDK4 *and *P-p38* expression decreased (P<0.01, P<0.05) in silica-induced malignant transformation cells compared with the control group. *P-ERK*, *P-JNK* and *Cyclin D1* expression increased (P<0.01, P<0.01, P<0.05) in B(a)P-induced group compared with the control group. *P-ERK* and *P-JNK* expression decreased (P<0.01), while *P-p38*,* Cyclin D1* and *CDK4* expression increased (P<0.05, P<0.05, P<0.01) in B(a)P-induced group compared with the silica-induced group.

**Conclusion:**

MAPK and cyclin D1/CDK4 activation expressed differently in human embryo lung fibroblasts malignant transformation induced by silica and benzopyrene.

## Introduction

DNA replication and mitosis of eukaryotic cells followed by the cell cycle of G1→S→G2→M, and ultimately cell proliferation (Qie, 2016). Orderly cell cycle is controlled by cyclins, CDKS and CDKI periodic accumulation and degradation (Du, 2006). Cyclin D1 and CDK4 are important enzymes regulating cell from G1 to S phase transformation (Chen et al., 2017).

The major molecular events of chemical carcinogenesis include gene mutation, telomere regulation, cell cycle regulation, cell apoptosis and the occurrence of tumor (Cohen and Arnold, 2011). The international agency for research on cancer (IARC), headquartered in Lyon, France, divided chemical substances into 4 levels based on human carcinogenic information and experimental animal carcinogenic data (Han et al., 2014): sufficient evidence of carcinogenicity, limited evidence of carcinogenicity (Class IA carcinogen), insufficient evidence of carcinogenicity (Class IB carcinogen), lacked evidence of carcinogenicity (Class II carcinogen). IARC claims that the cancer cases will increase 75% all over the world by the end of 2030. Silica and B(a)P were both classified as carcinogens. Silica was listed as a human carcinogen by IARC in 1997. B(a)P was a former carcinogen that required metabolic activation to form the final activated substance, which has also been listed as a human carcinogen by IARC (Park et al., 2015; Cui et al., 2017). But now, the carcinogenic mechanisms of silica and B(a)P are unclear.

Quartz is one of the most widely distributed minerals on the earth’s surface. It is the main form of silica in the earth’s crust (Diab et al., 2017). Because of optical, thermal and chemical durability, it is widely used in optical communication systems, foundry, ceramics, refractories, metallurgy, construction, and chemical industries as a heat-resistant glass and semiconductor materials (Cullinan et al., 2013). Content of free silica in quartz might be as high as 99%. Silica would cause lung inflammation for short term exposure and silicosis and lung cancer for chronic exposure (Steenland and Ward, 2014; Jia et al., 2010; Li et al., 2017). Silicosis is one of the serious occupational disease in China (Jia et al., 2011). Coal mine workers, quarry workers, tunnel workers, foundry and pottery workers and construction workers are at high risk of developing silicosis. Studies have shown (Li et al., 2016) that 200 µg/ml quartz can make HELF cell proliferation. The proportion of S phase cells increased significantly, while that of G1 phase cells decreased. At the same time, silica can induce overexpression of cyclin D1 and *CDK4*, and induce the change of HELF cell cycle through the phosphatidylinositol 3-kinase (PI-3K)/AP-1 signaling pathway.

In the polycyclic aromatic hydrocarbon (PAHs) family, BaP is the most widely pollution and most carcinogenic (Wester et al., 2012). Studies have shown that lung cancer mortality would increase 5%, while benzopyrene content increased 1% in the living environment. B(a)P can lead to cell cycle acceleration, DNA repair time shortening, the possibility of malignant transformation increasing. Research (Shen et al., 2008) has shown that B(a)P or its active metabolites lead to many genes and proteins involved in biological processes, such as cell proliferation, energy metabolism, DNA synthesis. *Cyclin D1 *and *CDK4 *genes were involved in cell cycle process induced by B(a)P, and played a positive regulatory role (Ye et al., 2006). This is likely to be carcinogenic mechanism of B(a)P.

Mitogen activated protein kinase (MAPK) is a group of important regulatory proteins, which belongs to serine/threonine protein kinase, which is expressed in all eukaryotic cells. It includes three subfamilies of ERK, p38 and JNK (Jia et al., 2011). Activated MAPK can activate nuclear transcription factor and other protein kinases and other substrates, regulate the transcription of related genes. It participates in many physiological processes, such as cell growth, development, division and functional synchronization between cells. It plays an important role in the pathological process of malignant transformation of cells.

AP-1 is an important regulator of the anterior inflammatory response kinase. It is related to the occurrence and development of pneumoconiosis. It is also associated with cell proliferation and tumor promotion. The activation of AP-1 is regulated by MAPK. The activation of each member of the MAPK subfamily has a specific phosphorylation cascade (Kavya et al., 2017). ERK pathway regulates cell growth and differentiation. In comparison with ERK, JNK and p38 often play a weaker response to growth factor stimulation, and have stronger response to environmental stress signals. These activations often lead to apoptosis.

This study aims to explore Cyclin D1, CDK4 and difference of cell cycle adjusted by MAPK signal transduction pathway in silica and B(a)P-induced malignant transformation of human embryonic lung fibroblasts.

## Materials and Methods

*Grouping*

Human embryonic lung fibroblast (HELF) was purchased from the cell room, basic research institute, Chinese Academy of Medical Sciences. The subjects will be divided into three groups, namely control group, silica induction group, B(a)P induction group.

We choose 200 µg/cm^2^ exposure of quartz inducing malignant transformation of cells, referencing the dose of experimental results of quartz cell toxicity. We screened silica-induced malignant transformation of human embryonic lung fibroblasts (Liu et al., 2004). HELF were exposed to B(a)P at a concentration of 100 µmol/L, per 24h and three times, by the interval of 24h. After 12 weeks of culture, we obtained malignant transformation of HELF (Ye et al., 2008). The malignant transformation cells were frozen in our lab. They should be cultured in serum-free culture medium for 72h before experiment.

We detected ERK, JNK and p38 phosphorylation levels and total protein, and expression levels of *CDK4 *and *cyclin D1*. Each experiment was conducted at least thrice in duplicate. The mean value was as the index of detection. 

*Reagents and Instruments*

ERK phosphorylation antibody, ERK total antibody, JNK phosphorylation antibody, JNK total antibody, p38 phosphorylation antibody and p38 total antibody were purchased from cell signaling technology Inc. Horseradish peroxidase-conjugated secondary antibody was purchased from Jackson Inc. Actin antibodies, cyclin D1 and CDK4 monoclonal antibody were purchased from Santa Cruz Biotechnology Inc. Protein quantification kit was from Nanjing jiancheng Bioengineering Institute. Blocking Reagent was the AMRESCO company’s products. Skimmed milk powder was purchased from Ding multinational companies. Nickel chloride, Tween-20 were obtained from Huamei company. Developer and fixer were purchased from the Lucky company’s. X-rays films were available from Kodak Company. Glycine was from Sigma.

Electrophoresis apparatus was from Bio Rad. UV spectrophotometer was purchased from Shimadzu. -70^o^C refrigerator was obtained from Hitachi company. CO_2 _incubator available from Ravco companies. High-speed centrifuge was from Hitachi company. Rotary mixer was purchased from Scientific Industries INC Company. Filter was obtained from the Gelman company.

*Expression levels of Cyclin D1, CDK4 and ERK, p38, JNK phosphorylation and total proteins.*

The subjects were the malignant transformation HELF induced by quartz and benzopyrene. We detected the expression levels of *ERK*, *JNK*, *p38* phosphorylation and total proteins, and cyclin D1, CDK4 by using western blot (WB) method. Kodak Digital Science 1D (KDS1D) image analysis software was used to analyze gray scale. The average light intensity detection of strips represented the level of protein expression. The larger average light intensity the more protein expression. 

*Statistical Analysis*

In this study, the SPSS16.0 statistics software was used for data processing, and the statistical results were expressed as mean with the standard deviation. One-way ANOVA was used among the experimental groups. We used the LSD test to further compare the results between two groups. P≤0.05 was considered as statistical significance.

## Results

*Cyclin D1 and CDK4 in malignant transformation HELF induced by silica and B(a)P*

**Figure 1:**
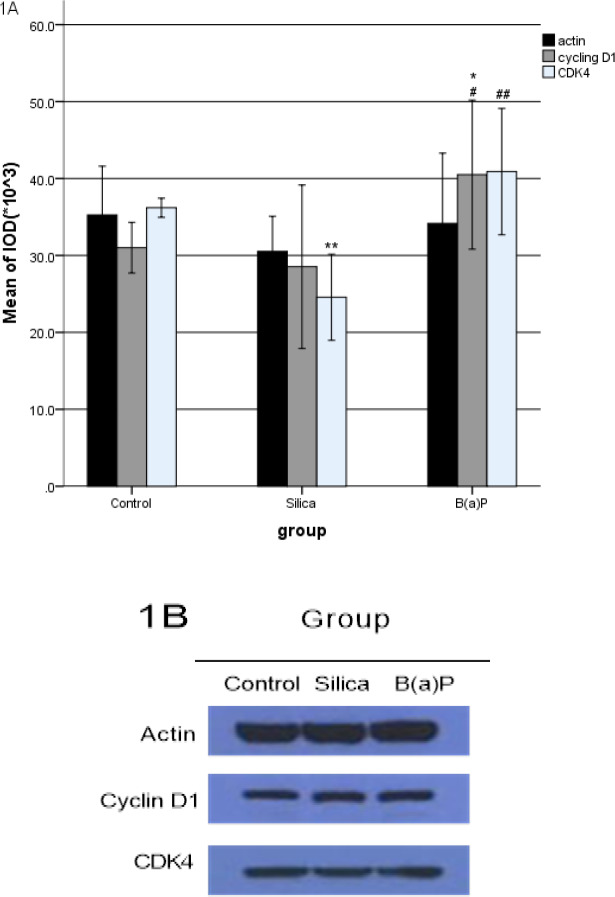
The Cyclin D1 and CDK4 Expressed in Malignant Transformation HELF Induced by Silica and B(a)P

Cyclin D1 and CDK4 phosphorylation levels in malignant transformation HELF induced by silica and B(a)P were shown in Figure 1 and Table 1. The reference protein *ACTIN* expression levels have no difference among the groups. But *Cyclin D1* and *CDK4* expression levels were differences among the groups. *Cyclin D1* expression was higher significantly in B(a)P transformation group than the control group and silica transformation group.* CDK4* expression was lower in silica transformation group than the control group, while higher in B(a)P transformation group than the silica transformation group. 

*ERK, p38 and JNK in malignant transformation HELF induced by silica and B(a)P*

**Figure 2:**
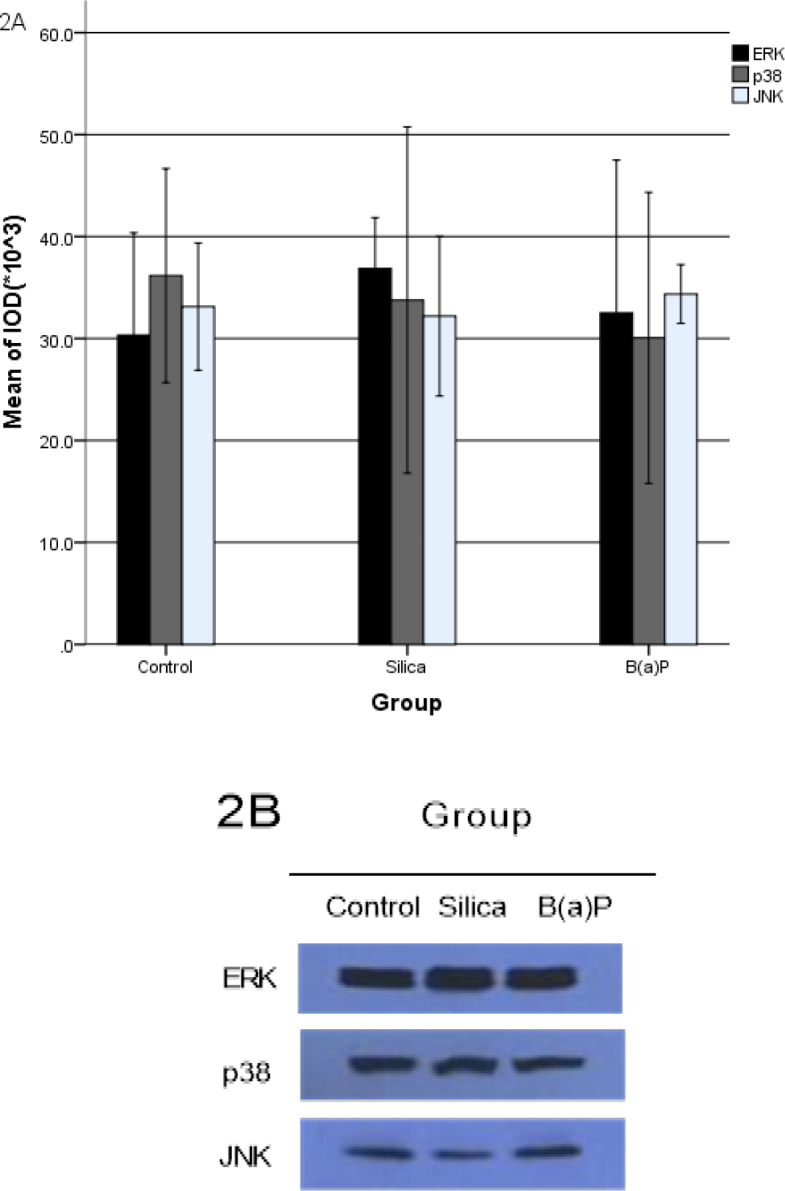
The Total Levels of ERK, p38, JNK Expressed in Malignant Transformation HELF Induced by Silica and B(a)P

ERK, p38 and JNK in malignant transformation HELF induced by silica and B(a)P were shown in Figure 2 and Table 2. Compared with the control group, total level of *ERK*, *p38* and *JNK* expression have no difference in malignant transformation HELF induced by silica and B(a)P. 

*ERK, p38 and JNK phosphorylation level in malignant transformation HELF induced by silica and B(a)P*

The phosphorylation state of *MAPK* subfamilies is the activation state. From the statistical results, expression of phosphorylation in *MAPK* subfamilies were different. Compared with the blank control group,* P-ERK* and *P-JNK* expression were higher significantly in silica transformation group and B(a)P transformation group, while *P-p38* expression was significantly lower in silica transformation group. *P-ERK* and *P-JNK* expression were significantly lower in B(a)P transformation group than the silica transformation group,* P-p38* expression was higher in B(a)P transformation group. 

**Table 1:** Cyclin D1 and CDK4 in Malignant Transformation HELF Induced by Silica and B(a)P ( ±s)

Group	N	ACTIN	Cyclin D1	CDK4
Control group	3	35.27±3.17	31.00±1.65	36.20±0.62
Silica transformation group	3	30.53±2.27	28.53±5.32	24.57±2.80**
B(a)P transformation group	3	34.17±4.56	40.50±4.84*#	40.90±4.11##
*F*		1.534	6.601	25.347
*P*		0.29	0.031	0.001

Note: *P< 0.05, compared with control group.#P < 0.05, compared with silica transformation group; **P< 0.01, compared with control group; ##P < 0.01, compared with silica transformation group.

**Figure.3:**
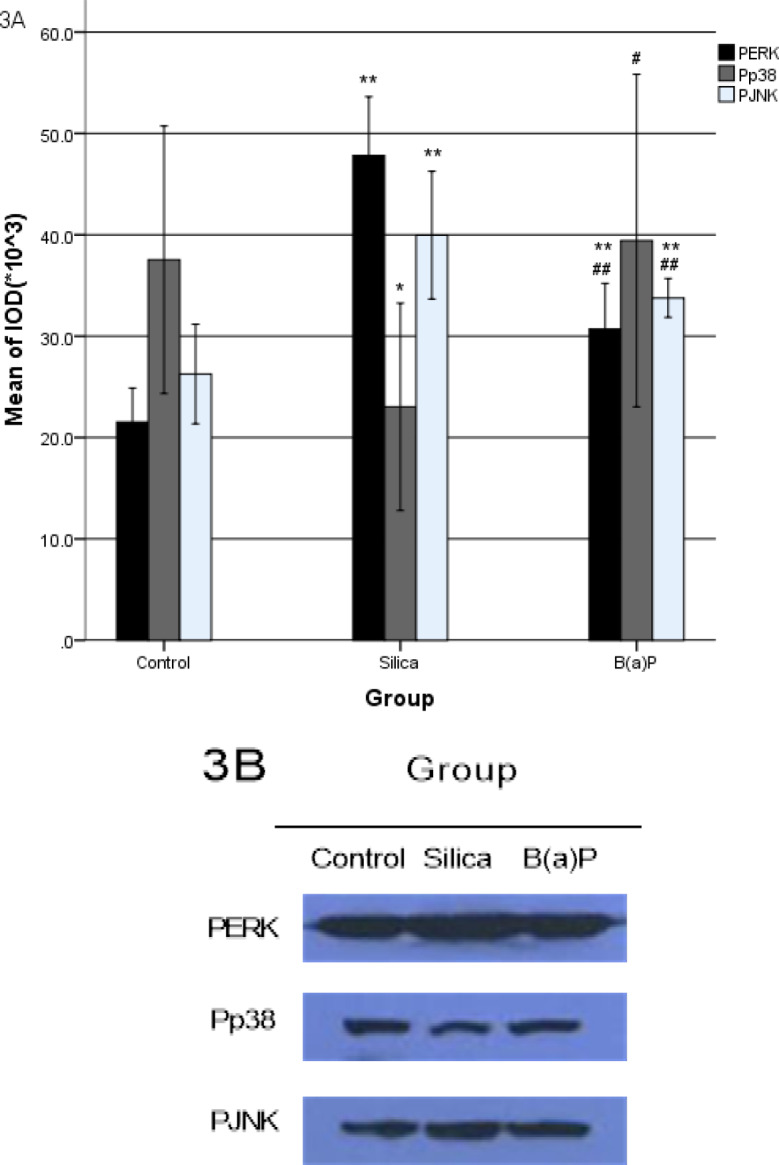
The Phosphrylation of ERK, p38, JNK, Cyclin D1 and CDK4 Expressed in Malignant Transformation HELF Induced by Silica and B(a)P Cyclin D1 and CDK4 (Figure 1), ERK, p38 and JNK (Figure 2), P-ERK, P-p38 and P-JNK (Figure 3) were expressed in malignant transformation HELF induced by silica and B(a)P. Each experiment was conducted at least thrice in duplicate. Error bars indicate standard deviation(SD). *P< 0.05, compared with control group. **P< 0.01, compared with control group. #P< 0.05, compared with silica transformation group. ##P < 0.01, compared with silica transformation group

**Table 2:** ERK, p38 and JNK in Malignant Transformation HELF Induced by Silica and B(a)P ( ±s)

Group	N	ERK	P38	JNK
Control group	3	30.33±5.02	36.17±5.25	33.13±3.12
Silica transformation group	3	36.87±2.49	33.77±8.49	32.20±3.92
B(a)P transformation group	3	32.50±7.50	30.07±7.14	34.37±1.44
*F*		1.137	0.564	0.392
*P*		0.381	0.596	0.692

**Table 3:** ERK, p38 and JNK Phosphorylation Amount in Malignant Transformation HELF Induced by Silica and B(a)P ( ±s)

Group	N	P-ERK	P-p38	P-JNK
Control group	3	21.53±1.68	37.53±6.60	26.27±2.46
Silica transformation group	3	47.83±2.89**	23.03±5.12*	39.97±3.15**
B(a)P transformation group	3	30.70±2.25**##	39.43±8.21#	33.77±0.96**##
*F*		98.661	5.286	25.108
*P*		0.001	0.047	0.001

Note: *P< 0.05, compared with control group.#P < 0.05, compared with silica transformation group.**P< 0.01, compared with control group. ##P < 0.01, compared with silica transformation group.

## Discussion

Previous studies Shen et al., (2006) showed that there were differential effects of ERKs, JNKs and p38, as well as their downstream transcription factor AP-1, in regulation of expression of *cyclin D1* and *CDK4* and cell cycle alternations in malignant transformation HELF induced by silica. Inhibition of ERKs activation by AG126, AP-1 by curcumin, and JNKs by SP600125 could reduced the induction of cyclin D1 and CDK4, whereas inhibition of p38K by SB203580 did not show inhibitory effects on S-HELF. These results demonstrated that ERKs and JNKs, but not p38, are responsible for induction of cyclin D1 and CDK4.

In this study, ERK, JNK were activated in malignant transformation HELF induced by silica and B(a)P. p38 was inhibited in silica transformation HELF, but not in B(a)P transformation HELF. The MAPK family is involved the process of malignant transformation induced by silica and B(a)P, but the specific process is not the same. Compared with the control group, *cyclin D1* expression increased in B(a)P-induced malignant transformation cells-while CDK4 expression was decline in silica transformation group.

The eukaryotic cells are organized in an orderly manner of replication and mitosis according to G1→S→G2→M phase, and ultimately to achieve cell proliferation. Throughout the cell cycle, the regulatory factor of G1 phase is related to malignant transformation closely. Cyclin D1 is a major cell cycle factor of G1 phase, which is closely related to cell growth, differentition and tumorigenesis (Jiao et al., 2008). Cyclin D1 and CDK4 are important enzymes in regulating cells from G1 to S phase. *Cyclin D1* expression increased (P<0.05) in B(a)P-induced malignant transformation cells compared with the control group. *CDK4 *and *Cyclin D1* expression increased (P<0.01, P<0.05) in B(a)P-induced group compared with the silica-induced group. It was proved that mechanism of malignant transformation in human embryo lung fibroblasts induced by quartz and benzopyrene was different.

Malignant transformation HELF induced by silica was mainly through down-regulation cyclins, while malignant transformation HELF induced by B(a)P was through up-regulation cyclins. It may be related to the degree of phosphorylation of p38. The expression of *cyclins* was inhibited after the activity of p38 was inhibited. It also may be related to the induced dose of silica and B(a)P. Inner link between p38 and cyclins, the dose-response relationship in malignant transformation HELF induced by silica and B(a)P need further study. 

According to Table 1, *Cyclin D1* expression was higher significantly in B(a)P transformation group than the control group and silica transformation group. In addition, *CDK4 *expression was higher in B(a)P transformation group than the silica transformation group, and it was lower in silica transformation group than the control group. Cyclin D1 and CDK4 were positive adjustment factor of cell cycle. They can regulate cell from G1 to S phase transformation to promote cellular processes. This showed the ability of malignant transformation in B(a)P was stronger than silica.

In summary, the activated subfamilies of MAPK, P-ERK, P-p38 and P-JNK, involved in the process of malignant transformation HELF induced by silica and B(a)P. Mechanism is different in malignant transformation HELF induced by silica and B(a)P. Compared with silica, the ability of malignant transformation inducted by B(a)P is more stronger.

## Abbreviations

MAPK, mitogen-activated protein kinase; AP-1, activator protein-1; ERK, extracellular signal-regulated kinase; JNK, c-Jun NH2-terminal amino kinase; CDK4, cyclindependent kinase 4; B(a)P, Benzo(a)pyrene; HELF, human embryonic lung fibro blasts; IARC, International Agency for Research on Cancer; WB, Western blot.

## Funding Statement

This work was supported by the grants of Natural Science Foundation of Hebei Province[H2017209195].
